# The Cantril Ladder as a Measure of Well-Being and Life Satisfaction Among Refugee Youth Experiencing Symptoms of Post-Traumatic Stress

**DOI:** 10.1007/s10903-023-01563-5

**Published:** 2023-10-26

**Authors:** Salma Elmukashfi Eltahir Mohammed, Georgina Warner

**Affiliations:** Child Health and Parenting (CHAP), Department of Public Health and Caring Sciences, Uppsala University, Box 564, BMC, Husargatan 3, Uppsala, Sweden

**Keywords:** Cantril Ladder, Well-being, Life satisfaction, Refugee youth, Post-traumatic stress

## Abstract

Given the number of refugee youth whom require mental health support, there is a need to provide community-based interventions that can be easily scaled-up at a low cost. Yet, safety procedures associated with community-based intervention require careful consideration. The Cantril Ladder is a visual scale used to assess life satisfaction. It could be a useful tool to track the well-being of participants throughout an intervention. However, concerns have been raised about the validity of single-item life satisfaction measures and it is recommended they are tested when used in specific populations. This is particularly relevant to the refugee youth population who experience ongoing stress due to concern for family, friends, housing, and schooling and whose perceptions of life satisfaction may differ to the cohorts the Cantril Ladder has previously been tested with. The purpose of this study was to explore the validity of the Cantril Ladder as a measure of well-being and life satisfaction in refugee youth experiencing post-traumatic stress symptoms by exploring the relationship between how the youth scored on the Cantril Ladder with their scores on measures of depression and self-efficacy. Hierarchical multiple linear regression was applied to self-reported survey data in order to test how refugee youth experiencing post-traumatic stress symptoms (n = 51) score on the Cantril Ladder compared with Patient Health Questionnaire-9 (PHQ-9) and General Self Efficacy Scale (GSE) scores. The mean Cantril Ladder score in the present sample was 5.33 (SD = 2.77). The PHQ-9 and GSE total scores together explained 19.1% of the variability in the Cantril Ladder score. The Cantril Ladder showed moderate concurrent validity with validated measures of depression and self-efficacy. These findings have important implications for intervention programs designed to support refugee youth experiencing post-traumatic stress, as the Cantril Ladder offers a promising way to track well-being throughout the program as part of a wider safety protocol procedure. Additional research is required to not only confirm these findings, but also to test the face validity of the Cantril Ladder for a more complete validation of life satisfaction.

## Background

Research indicates that asylum-seeking and refugee children and youth are at increased risk of poor mental health outcomes [1–3]. They are affected by high levels of post-traumatic stress disorder (PTSD), but also by anxiety and depression disorders [4, 5]. A systematic review and meta-analysis demonstrated that refugee children and youth who have resettled in high-income western countries are vulnerable to mental disorders [6]. Indeed, refugee children and youth present with up to ten times more prevalence of PTSD and depression rates compared with the general population [6]. Previous research in Sweden has found no significant relation between the prevalence of symptoms and country of origin, type of living arrangements, length of journey, or time spent in Sweden, implying that the risk of PTSD is universal in refugee youth [7].

Given the number of refugee youth whom require support, there is a need to provide community-based interventions that can be easily scaled up at a low cost. However, the necessary safety processes associated with this approach require attention. This article details a small-scale study conducted as part of the randomised controlled trial evaluation of a community-based intervention called Teaching Recovery Techniques (TRT) with refugee youth in Sweden [8, 9]. Predominantly based on trauma-focused cognitive behavioural therapy, TRT is a brief group-based intervention delivered over five to seven weekly sessions by professionals working with children whom have attended a three-day training [10]. As the youth are at increased risk for suicide and the TRT group leaders may have no previous psychological training, there is a need for a ‘safety protocol’. This is a structured way to ensure group leaders are regularly checking on the well-being of the youth and have advice on where to refer them if they are suicidal. In Sweden, TRT group leaders administer the Cantril Ladder [11] at each TRT session. If a TRT group participant indicates ‘suffering’ on the Cantril Ladder by scoring four or below, the group leader is instructed to use the Columbia Suicide Severity Rating Scale Screen Version [12] with the participant. The safety protocol specifies when and where to refer to if the youth needs further support and service tools and has been modified to align with service availability in local areas.

### The Cantril Ladder

The Cantril Ladder is a visual scale used to assess general well-being and life satisfaction [11]. Respondents, such as the youth receiving TRT, are presented with a picture of a ladder numbered from zero to ten, where zero reflects the worst life satisfaction and ten reflects the best life satisfaction. The youth are asked to think about their life at present and place themselves on the ladder. A score equal to or below four is expressive of ‘suffering’ and seven or above ‘thriving’. In other words, a higher score indicates greater well-being and life satisfaction. Therefore, the administration of the Cantril Ladder is simple and can offer a quick and easy way to assess general well-being at every TRT session to inform the safety protocol without a significant investment of time for either the participant or the group leader. The scale is considered a valid and reliable measure of life satisfaction and psycho-social health among the youth of ages 10–17 years [13, 14].

However, concerns have been raised about the validity of single-item life satisfaction measures. Although there is evidence that single-item measures have relatively high reliability, their validity might be low or weaker when compared with multiple-item scales [15]. The main argument is that single-item measures are necessarily narrow in focus and may not be able to capture the breadth that can be assessed with multiple items [16]. Thus, single items perform as well as multi-item scales only under particular conditions. For this reason, the use of single-item measures should be limited to special circumstances [17]. Hence there is a general argument that the validity of such measures should be tested when used in specific populations. This is particularly relevant to the refugee population whom experience ongoing stress due to concern for family, friends, housing, and schooling, but their emphasis is also influenced by political, economic, social and cultural structures in their new environment [18]. This indicates that, as a group, they might have different perceptions of life satisfaction.

### The Relationship Between Life Satisfaction and Depression

Life satisfaction is strongly and negatively correlated with negative psychological problems such as depression, social stress, and suicide [19–24]. Low life satisfaction has even been shown to predict mental health problems, such as depression, anxiety, as well as suicide [19, 25]. On the other hand, higher depression symptoms more strongly predict decreases in life satisfaction than the reverse [26]. Some researchers have suggested that clinical depression is the loss of subjective well-being, which can be measured with expressions of life satisfaction [27]. Other studies show that depression is related to identity and life satisfaction, where lower levels of depression are associated with higher levels of life satisfaction [21, 28]. Therefore, adolescents with less emotional stability would tend to feel less life satisfaction and more depressive symptoms, which in turn would facilitate greater suicidal ideation [21].

### The Relationship Between Life Satisfaction and Self-Efficacy

Findings from previous correlational analysis have shown life satisfaction to be positively associated with self-efficacy [29]. Self-efficacy provides an important advantage not only by increasing life satisfaction but also in terms of psychological and social development [30]. Previous research suggested that raising self-efficacy of emerging adults is vital for their healthy development and life satisfaction [31]. The positive relationship between self-efficacy and life satisfaction can be understood by the fact that the people with high self-efficacy have the ability to overcome stressful life events because these people are reported to have the attitude “I can do this” and hence self-efficacy can facilitate coping and mitigate the negative outcome of stress on life satisfaction [32]. Therefore, any increase in self-efficacy was associated with increase in life satisfaction. On the contrary, the individuals with low self-efficacy believe that the things they will do are harder than they are in reality; such a thought increases the anxiety and stress which have been shown to be associated with a decrease in life satisfaction [32].

### Aim

The purpose of this study was to explore the validity of the Cantril Ladder as a measure of well-being and life satisfaction in refugee youth experiencing post-traumatic stress symptoms by exploring the relationship between how the youth scored on the Cantril Ladder with their scores on measures of depression and self-efficacy. The hypothesis for the study was that refugee youth depression and self-efficacy scores could explain variance in Cantril Ladder total scores; therefore, the Cantril Ladder would function with refugee youth experiencing post-traumatic stress symptoms as with a general population of adolescents.

## Methods

### Design

The study design was a cross-sectional self-report survey based on data derived from pre-intervention data collection within ongoing trials of TRT in Sweden [8, 9].

### Setting

The participants were recruited from schools and healthcare clinics in various regions across Sweden, including Linköping, Uppsala, Östersund, Västerås, and Gävle between April 2019 and March 2020.

### Participants

The study sample size was derived from the available pre-intervention data in the ongoing trials. The sample included 51 eligible participants (41 male and 10 female) between 12 and 20 years of age (M = 17.5 years; SD = 2.06). Retrospective power analysis indicated the sample size was sufficient for the analyses performed [33]. According to the trial inclusion criteria, all included youth screened positive for PTSD on the Child Revised Impact of Events Scale-8 (total score ≥ 17); they had all spent five years or less in Sweden; were interested in participating in the TRT intervention; and were not receiving ongoing treatment where a therapist advised against involvement in the TRT intervention. The youth were from different countries of origin and completed the survey in various languages, including Swedish [19], Tigrinya [11], Dari [10], Arabic [6], Somali [4], and English [1].

### Materials

#### The Children’s Revised Impact of Event Scale-8 (CRIES-8)

The CRIES-8 [34] is a brief self-report measure designed to screen children aged 8 years and above at risk for PTSD. It contains eight items, four focused on symptoms of Avoidance (e.g. *Do you try to remove it from your memory?*) and four on Intrusion (e.g. *Do you think about it even when you don’t mean to?*). The respondent is asked to indicate how often they experience each symptom (*Not at all = 0; Rarely = 1; Sometimes = 3; Often = 5*). The total score ranges from 0 to 40, with a cut-off score of 17 indicating PTSD.

#### The Cantril Ladder

A visual scale used to assess general well-being and life satisfaction [11]. Participants were presented with a picture of a ladder with the following description: *Here is a picture of a ladder. Suppose the top of the ladder represents the best possible life for you and the bottom of the ladder the worst possible life. Where on the ladder do you feel you stand at the present time?”* Total scores range from 0 to 10 with a higher score indicating greater well-being and life satisfaction. Three levels of life satisfaction were defined, ranging from low (0–4), average [5–7] to high [8–10].

#### Patient Health Questionnaire-9 (PHQ-9)

The PHQ-9 is a nine-item self-administered diagnostic instrument for measuring the severity of depression [35]. The PHQ-9 total score ranges from zero to 27. Each of the nine items (e.g. *Feeling down, depressed, or hopeless*) is assessed according to the frequency of their occurrence during the past two weeks (*Not at all* = 0, *Several days* = 1, *More than half the days* = 2, *Nearly every day* = 3). The total score is calculated by finding the sum of the nine items. A score of 5, 10, 15, and 20 signifies mild, moderate, moderately severe, and severe symptoms, respectively. A score of 20 to 27 required an immediate referral to a mental health specialist (96,100). The instrument has become increasingly used in both clinical practice and research due to its diagnostic validity, brevity, and ease of scoring. The scale has been validated and shown a Cronbach’s α = 0.86 and 0.89 in two different samples respectively and had test-retest reliability r = 0.84, which represents high internal consistency [35]. Adequate sensitivity of 0.71 to 0.87 and high specificity of 0.88 and 0.95 have been found for PHQ-9 ≥ 10 [36]. PHQ-9 was also found to be a valid measure of treatment outcomes, with a change in scores of five suggested to reflect a clinically relevant change [37].

#### General Self Efficacy Scale (GSE)

The GSE is a 10-item self-report instrument [38]. It is designed to assess the strength of respondent’s beliefs and judgement of their own ability to respond to and deal with difficult situations (e.g. *I can always manage to solve difficult problems if I try hard enough*) [39]. Therefore, the scale can predict coping and adaptation after experiencing stressful life events. The instrument has four-point scale responses, and the sum of all the ten items yield a total score range from 10 to 40, with a higher score indicating more self-efficacy [39]. Responses are rated according to how accurate the item statement is for the responder from one to four (*1 = not at all true, 2 = hardly true, 3 = moderately true, 4 = exactly true).* In samples from 25 nations, this scale was reliable and valid across different cultures with Cronbach’s alpha ranging from 0.75 to 0.91 [40].

### Data Collection

Outcome data were collected using a secure online platform (Qualtrics). For analysis, data were exported and inputted into a Statistical Package for the Social Sciences (SPSS) file. Anonymous participant identity numbers were used. Then the data were secured in a server at Uppsala University and automatically backed up. All procedures have complied with the current regulations on personal data management.

### Analysis

IBM SPSS Statistics version 26 was used to perform all the statistical analyses. The full sample was summarised by descriptive statistics such as mean and standard deviation for numerical variables and frequency (percentage) for categorical variables. Hierarchical multiple linear regression was applied to the data in order to test the Cantril Ladder total score against the PHQ-9 and the GSE scores. Models were built by adding one variable at each stage of the modelling procedure in order to assess the effect of each new variable on the relationship between life satisfaction and the exploratory variables. The PHQ-9 total score was entered first to determine if it contributes a unique proportion of variance to the life satisfaction scores. To control for demographic variables, age, gender and language were also entered in the first preliminary regression, but were not found to be significantly related to life satisfaction total scores and so were excluded from the final analyses. The regression equations met assumptions of normality, linearity, and homogeneity of variance, and no problematic multicollinearity was apparent. The linear regression assumption was verified by calculating the residuals and prove their normalities, homoscedasticity and independence. A scatter plot was drawn between the dependent and each of the independent variables to check for linearity in the association between the two variables and to detect the presence of outliers that may not have been identified during the data management procedures. Kolmogorov-Smirnov test was performed to test for normal distribution of each of the variables in the model. Linear regression analyses were performed in order to establish a correlation between variables and to test the null hypothesis that there was no relationship between the independent variables and the dependent variable. The regression coefficients, statistical significance of regression model (*t* value), and the multiple correlation coefficient (Adjusted *R*^2^) were calculated.

### Ethics Approval and Consent to Participate

The data were utilised from an ongoing project, which received clearance from the Uppsala Regional Ethics Committee (Dnr. 2018/382). Youth were informed about the purpose, content, and eligibility criteria of the study. The youth, and their legal guardian if the youth was under 15 years old, were informed that the data would be treated confidentially. Moreover, the results will be reported and published anonymously, and it will not be possible to recognise any individual or assign any information to them. Youth were offered an incentive (shopping vouchers valued at 100 Swedish krona) to compensate for their time completing the survey. The participants were made aware that participation was voluntary, and they could withdraw at any time. All existing data could be retained unless a youth/legal guardian asked for withdrawal and removal of the provided data. At this situation, they would be informed that this is possible up to the point that the data was analysed. Also, they were informed that if they disclosed anything concerning their safety then a safety protocol, whereby specialist services are contacted, would be implemented.

## Results

### Sample Characteristics

The mean age of the 51 participants was 17.5 years (SD = 2.06) The majority of the youth were over 16 years of age (72.5%). As a group, they assessed their life satisfaction on the Cantril Ladder as 5.33 (SD = 2.77) out of 10 points on average. The mean score for depression was 12.08 (SD = 6.50) out of 27, which indicates a moderate level of severity. The mean score for self-efficacy was 27.31 (SD = 6.59) out of 40.

### Regression Analysis

All the outcome measure variables were found to have good Cronbach’s alpha (α) scores that were higher than the threshold value of 0.70. This consistency coefficient indicates a high level of internal consistency for the scales used in the present sample. There was no association between gender and the outcome measure variables. While analyses initially indicated a relationship with age, it was excluded during the regression analysis due to collinearity.

**Fig. 1:**
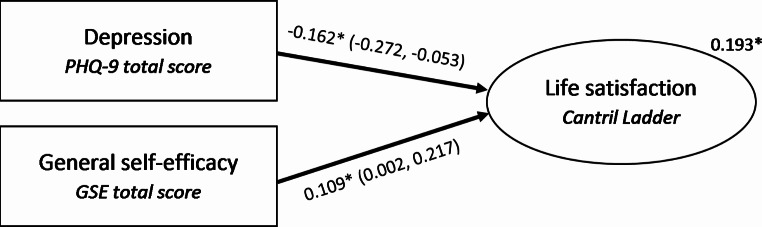
Visual depiction of regression analysis results. Shown above the arrows are regression coefficients (95% confidence intervals), and above Life satisfaction the adjusted squared multiple correlation (R^2^). *p < 0.05

The linear regression established that PHQ-9 total score could significantly explain Life satisfaction (*R* = 0.398, R^2^ = 15.9, F (1,49) = 9.232, p = 0.004) and alone accounted for 15.9% of variability in Cantril Ladder total score. When GSE total score was added to the model, the results of the regression indicated that the two variables explained 19.3% of the Cantril Ladder total score variance (Adjusted R^2^ = 0.193, F (1,48) = 6.997, p = 0.002) (Fig. 1). The GSE total score explained 6.7% of the variability in the Cantril Ladder total score in the presence of PHQ-9 total score.

Concerning the explanatory variables, PHQ-9 total score showed a significant but negative association with the Cantril Ladder total score. In contrast, the GSE total score was positively and significantly associated with the Cantril Ladder total score. For each score increase in PHQ-9 total score, there is a decrease in the Cantril Ladder total score of 0.162 while holding the other variable constant (B = -0.162, 95% CI; -0.272, -0.053). On the other hand, for each point increase in GSE total score, there is an expected increase in the Cantril Ladder total score by an average of 0.109 while taking in to account other variables in the model (B = 0.109, 95% CI; 0.002, 0.217). The influence of PHQ-9 total score was found to be dominant over the influence of the GSE total score (Δ*R*2 = 0.159, *p* = 0.004).

## Discussion

Task shifting of psychological support from clinical to community settings to meet the mental health needs of refugee youth has presented a need for sound safety protocols in which ‘easy to administer’ tools can be used to track participant well-being and highlight those who need to be referred to more specialised support. Yet, there is a lack of evidence on the validity of brief well-being measures when used with refugee youth. This study endeavoured to explore the validity of the Cantril Ladder among refugee youth experiencing post-traumatic stress symptoms via uncovering the relationship between the respondent’s self-reports of their Cantril Ladder total score (life satisfaction), PHQ-9 total score (depression) and GSE total score (self-efficacy). It was assumed that life satisfaction would be negatively associated with depression and positively associated with self-efficacy. Hence participants’ total scores on depression and self-efficacy measures would explain their life satisfaction variabilities.

The mean Cantril Ladder score in the present sample was 5.33 (SD = 2.77). Data from the Health Behaviour in School-aged Children 2010 survey revealed that the mean Cantril Ladder total scores in 31 high-income countries in Europe’s and North America was 7.58 ± 1.89 [41]. Thus, the present data demonstrate less life satisfaction than the noted in the above cross-national data survey. Hence, refugee youth experiencing symptoms of post-traumatic stress require particular attention and, importantly, require a safety protocol during community-based interventions to safeguard them from the negative impact of low life satisfaction.

The key findings of the study analysis indicated that PHQ-9 total score and GSE total score together account in the model to explain 19.1% of the variability in the Cantril Ladder score. As expected, negative correlations were found between life satisfaction and measures of depression, which corresponds with findings from previous research [13, 26]. The current results also align with previous studies providing support for the idea that self-efficacy is positively associated with adolescents’ life satisfaction and quality of life [42–45].

When looking at the amount of variance explained, it could be interpreted as small, which could be attributed to the inaccurate methodological measurement of the dependent and independent variables as the data were derived from self-reports and the outcomes are subjective. However, Baumeister, Vohs, and Funder (2007) demonstrate that the use of self-report is validated and found it to be useful for assessing subjective well-being and cognitive evaluation, such as life satisfaction [46]. There were further limitations to the data collection such as limited information on the demographics of the sample, which could relate to life satisfaction. Despite the low explanatory power, the model was found to be significantly different from zero, thus, indicating the statistically significant explanatory power of the regression model. Therefore, the validity of the Cantril Ladder as a measure of life satisfaction in refugee youth experiencing symptoms of post-traumatic stress should not be underestimated. It can be deemed a satisfactory tool to track the well-being of refugee youth. However, it should be considered as the first stage in a more comprehensive safety protocol in which more detailed assessments of mental health are used when low well-being is indicated.

## Conclusions

In the current study, the Cantril Ladder showed moderate concurrent validity with validated measures of depression and self-efficacy. These findings have important implications for intervention programs designed to support refugee youth experiencing symptoms of post-traumatic stress, as the Cantril Ladder offers a promising way to track well-being throughout the program as part of a wider safety protocol procedure. Additional research is required to not only confirm these findings, but also to test the face validity of the Cantril Ladder for a more complete validation of life satisfaction. A qualitative research design would support this aim.

## Data Availability

The datasets analysed during the current study are available from the corresponding author on reasonable request.

## List of Abbreviations

CRIES-8: Child Revised Impact of Events Scale-8
GSE: General Self Efficacy Scale
PHQ-9: Patient Health Questionnaire-9
PTSD: post-traumatic stress disorder
TRT: Teaching Recovery Techniques
